# *In vitro* Interaction of *Pseudomonas aeruginosa* Biofilms With Human Peripheral Blood Mononuclear Cells

**DOI:** 10.3389/fcimb.2020.00187

**Published:** 2020-05-05

**Authors:** Esingül Kaya, Lucia Grassi, Arianna Benedetti, Giuseppantonio Maisetta, Carolina Pileggi, Mariagrazia Di Luca, Giovanna Batoni, Semih Esin

**Affiliations:** ^1^Department of Translational Research and New Technologies in Medicine and Surgery, University of Pisa, Pisa, Italy; ^2^Department of Transfusion Medicine and Transplant Biology, Pisa University Hospital, Pisa, Italy; ^3^Department of Biology, University of Pisa, Pisa, Italy

**Keywords:** peripheral blood mononuclear cells, cytokines, biofilm, *Pseudomonas aeruginosa*, human immune response to biofilm, natural killer cells

## Abstract

The human immune cell response against bacterial biofilms is a crucial, but still poorly investigated area of research. Herein, we aim to establish an *in vitro* host cell-biofilm interaction model suitable to investigate the peripheral blood mononuclear cell (PBMC) response to *Pseudomonas aeruginosa* biofilms. *P*. *aeruginosa* biofilms were obtained by incubating bacteria in complete RPMI 1640 medium with 10% human plasma for 24 h. PBMC obtained from healthy donors were added to preformed *P*. *aeruginosa* biofilms. Following a further 24 h incubation, we assessed (i) PBMC viability and activation; (ii) cytokine profiles in the supernatants; and (iii) CFU counts of biofilm forming bacteria. Cell-death was <10% upon 24 h incubation of PBMC with *P*. *aeruginosa* biofilms. PBMC incubated for 24 h with preformed *P*. *aeruginosa* biofilms were significantly more activated compared to PBMC incubated alone. Interestingly, a marked activation of CD56^+^CD3^−^ natural killer (NK) cells was observed that reached 60% of NK cells as an average of different donors. In the culture supernatants of PBMC co-cultured with *P*. *aeruginosa* biofilms, not only pro-inflammatory (IL-1β, IFN-γ, IL-6, and TNF-α) but also anti-inflammatory (IL-10) cytokines were significantly increased as compared to PBMC incubated alone. Furthermore, incubation of biofilms with PBMC, caused a statistically significant increase in the CFU number of *P. aeruginosa*, as compared to biofilms incubated without PBMC. In order to assess whether PBMC products could stimulate the growth of *P*. *aeruginosa* biofilms, we incubated preformed *P. aeruginosa* biofilms with or without supernatants obtained from the co-cultures of PBMC with biofilms. In the presence of the supernatants, the CFU count of biofilm-derived *P*. *aeruginosa*, was two to seven times higher than those of biofilms incubated without supernatants (*P* < 0.01). Overall, the results obtained shed light on the reciprocal interaction between human PBMC and *P*. *aeruginosa* biofilms. *P*. *aeruginosa* biofilms induced PBMC activation and cytokine secretion but, in turn, the presence of PBMC and/or PBMC-derived components enhanced the number of *P*. *aeruginosa* biofilm associated bacteria. This may indicate a successful bacterial defensive/persistence strategy against immune response.

## Introduction

*Pseudomonas aeruginosa* is an environmental Gram-negative opportunistic pathogen involved in a large spectrum of infections, especially in immunocompromised and hospitalized patients (Driscoll et al., 2007). Infections caused by *P. aeruginosa* include acute and chronic respiratory infections, hospital-acquired urinary tract infections, chronic infections of wounds, otitis, endocarditis, osteomyelitis, corneal infections, and systemic infections (Driscoll et al., 2007). *P. aeruginosa* plays a particularly critical role in cystic fibrosis (CF) patients, largely contributing to the rapid decline in pulmonary function and representing an important cause of morbidity and mortality (Malhotra et al., 2019). The bacterium is also frequently involved in infections associated to the use of intravascular catheters (Raad, 1998), urinary catheters (Vipin et al., 2019), prosthetic implants (Martinez-Pastor et al., 2009) and other medical devices that represent essential tools of the modern medicine (Tolker-Nielsen, 2014). The pathogenesis of many *P*. *aeruginosa* infections depends on a striking ability of the bacterium to form biofilms, complex bacterial communities adhering on a substrate, such as mucosal surfaces or invasive medical devices (Tolker-Nielsen, 2014). In biofilms, bacterial cells are typically embedded within a self-produced extracellular polymeric substance (EPS), primarily composed of polysaccharides, proteins, and extracellular DNA (eDNA) (Flemming and Wingender, 2010). EPS plays a crucial role in maintaining the biofilm architecture ensuring a highly hydrated microenvironment and favoring the interactions among bacterial cells. The biofilm mode of growth provides the bacteria with enormous advantages to establish an infection as renders them extremely recalcitrant to both antimicrobial treatment and immune responses (Batoni et al., 2016). In fact, the EPS acts like a barrier that hampers the diffusion of antibiotics as well as host immune cells. In this regard, it has been reported that antibodies or phagocytic cells at most enter the water channels intercalating the micro-colonies that constitute a mature biofilm (Costerton et al., 2003), but hardly penetrate into the deep layers of the micro-communities, especially when biofilms are grown under static conditions (Leid et al., 2002).

Despite the recognized clinical importance of biofilms, the human immune response against infectious biofilms is a research area that necessitates to be thoroughly investigated as the majority of immune research investigations have focused on bacteria in the planktonic state (Moser et al., 2017). A deep understanding of the complex interactions that establish between biofilm bacteria and the immune system may help in identifying new targets and strategies of immune intervention against biofilm-associated infections. In addition, quantitative measurements of the host responses to biofilms may serve as diagnostic tools or possible biomarkers for tracing the course of an infection (Moser et al., 2017; Campoccia et al., 2019).

The host response against *P*. *aeruginosa* biofilms is particularly complex and it is believed to involve the integrated activity of an array of cell types of both the innate and adaptive immune systems (Maurice et al., 2018). The dynamic interplay between *P*. *aeruginosa* and the host immune system is one of the major determinants of bacterium's pathogenicity and may shape the phenotype of chronic lung infections, which range from acute exacerbations to sub-clinical slowly progressing conditions characterized by the adaptation of the bacterium to the host and attenuated immune responses (Faure et al., 2018).

The *in vitro* human immune response to *P*. *aeruginosa* biofilms is a research area still poorly investigated. The cytotoxic effect exerted by the bacterium on a variety of host cell types is one of the main hurdles that hampers the study of the host immune response *in vitro* (Bishop et al., 1987; El-Housseiny et al., 2013). In addition, most of the *in vitro* studies conducted so far have investigated neutrophil or monocyte response to *P*. *aeruginosa* biofilms (Jesaitis et al., 2003; Walker et al., 2005; Ciornei et al., 2010), while little is known on the human peripheral blood mononuclear cells (PBMC) and biofilm interaction. In the present study, we sought to establish an *in vitro* model of *P*. *aeruginosa* biofilms and human PBMC co-culture suitable to assess immune responses (e.g., expression of activation markers, cytokine production) of different PBMC subsets. By finely tuning the experimental parameters, such as PBMC and bacteria numbers, incubation times, type of medium used for biofilm formation, we could obtain mature biofilms of *P*. *aeruginosa* in eukaryotic cell-compatible medium RPMI 1640 and maintain the *P*. *aeruginosa*-biofilm:PBMC co-culture for at least 24 h, with minimal cell death. Interestingly, the results obtained disclosed a reciprocal interaction between human PBMC and *P*. *aeruginosa* biofilms. *P*. *aeruginosa* biofilms induced PBMC activation and cytokine secretion but, in turn, PBMC and/or PBMC components enhanced the number of *P*. *aeruginosa* biofilm associated bacteria after 24 h of co-culture. These results suggest a successful bacterial defensive/persistence strategy in response to host immune response.

## Materials and Methods

### Bacterial Strains and Culture Conditions

The reference strain *P*. *aeruginosa* ATCC 27853 was used in the present study. In addition, the reference strain PAO1 (ATCC 15692) and two *P*. *aeruginosa* clinical strains isolated from abdominal fluid (PA-AF) and CF lung (PA-CF) at the Microbiology Unit of the University Hospital of Pisa, Italy were employed in some experiments. Stock cultures were prepared by growing bacterial strains in Tryptone Soy Broth (TSB) (Oxoid, Basingstoke, UK) at 37°C until mid-logarithmic phase. Bacterial suspensions were then aliquoted and stored at −80°C in the same medium until use.

### Biofilm Formation

An aliquot of frozen *P. aeruginosa* culture was thawed, diluted 100 times in TSB, and incubated at 37°C overnight (18 h) with shaking (600 rpm). A volume of 500 μl from the stationary phase culture was centrifuged at 4,000 × g for 5 min. at room temperature (RT) and the pellet was re-suspended in the same volume of complete medium consisting of RPMI 1640 (Euroclone S.p.A, Pero, Milan) supplemented with 10% heat-inactivated pooled human plasma and 2 mM L-glutamine (Euroclone). Preliminary experiments were performed to establish the optimal bacterial number ensuring biofilm formation in such medium. Addition of a 100 μl bacterial suspension containing ~1 × 10^6^ colony forming unit (CFU) per well to 96-well flat bottom plates (Euroclone) was found to be optimal for the experimental purposes. To this aim, initial bacteria suspension were diluted in complete RPMI medium to reach such bacterial density. To assess the exact number of initial bacteria used in each experiment, an aliquot was plated on Tryptone Soya Agar (TSA; Oxoid, Basingstoke, UK) plates for CFU count. The 96-well plates were incubated at 37°C for 24 h without shaking to allow biofilm formation. Following the incubation, biofilms were gently washed three times with phosphate buffered saline (PBS) to remove non-adherent cells and incubated for further 24 h in the absence or in the presence of PBMC (see below) in complete RPMI in humidified air containing 5% CO_2_. Following 24 and 48 h of incubation, biofilm-associated bacterial cells were mechanically detached from the bottom of the wells by scratching 60 s with a pipette tip, subjected to vigorous vortexing, serially diluted and plated on TSA (Oxoid). After an incubation of 24 h at 37°C the number of biofilm-associated bacteria (CFU/ml) was evaluated by CFU counting.

### Analysis of *P. aeruginosa* Biofilms Grown in Complete RPMI 1640 by Confocal Laser Scanning Microscopy (CLSM)

*P*. *aeruginosa* biofilms grown in complete RPMI were analyzed by CLSM as previously described (Maisetta et al., 2016; Di Luca et al., 2018). Briefly, biofilms were formed for 24 and 48 h in the same conditions as explained above (see “Biofilm formation”) but using 8 well ibiTreat polymer coverslips (ibidi GmbH, Gräfelfing, Germany). Following incubation, biofilms were gently washed with sterile MilliQ water (Millipore), and stained with green fluorescent Syto9 (live bacteria) and red fluorescent propidium iodide (dead bacteria) (Filmtracer™ LIVE/DEAD™ Biofilm Viability Kit, Thermo Fisher Scientific). Stained samples were observed under TCS SP5 II (Leica Microsystems Srl, Buccinasco, Milan) confocal microscope (interfaced with a 488 nm Argon laser), using a 60 × 1.25 NA water immersion objective. For each sample, the entire well surface and depth were scanned. 10 μm Axial stacks in the Z plane, with a slice thickness of 1 μm, were taken through representative areas of biofilm.

### PBMC:Biofilm Co-culture

Peripheral blood (buffy coat) from healthy subjects was obtained from the donors attending the Transfusion center of Pisa University Hospital or from healthy volunteers. An informed consent was obtained from each donor. The study was conducted in accordance with the Declaration of Helsinki, and the protocol was approved by the local Ethical Committee (Protocol 34743, 28/06/2018). PBMC were isolated by standard gradient protocol as described previously (Esin et al., 1996). In brief, blood was diluted with PBS (1:1 ratio) containing 10% sodium citrate (v/v), layered on a density gradient (Lymphoprep, Cedarlane, Canada), and centrifuged at 160 × g for 20 min at RT. Following centrifugation, supernatants were removed, without disturbing the mononuclear layer at the interface, to eliminate platelets. After a further centrifugation at 800 × g for 20 min, PBMC were collected from the interface. After three washes with RPMI, PBMC were resuspended in complete RPMI (RPMI 1640 added with 10% heat inactivated autologous plasma and 2 mM L-glutamine). Preformed biofilms (24 h-old) of *P. aeruginosa* were gently washed three times with PBS to remove non-adherent bacteria and 200 μl of PBMC suspension (2 × 10^6^ cells/ml) was added to *P*. *aeruginosa* biofilms (i.e., 4 × 10^5^ PBMC/well). PBMC:biofilm co-cultures were incubated at 37°C in 5% CO_2_ for 24 h. PBMC alone were used as a negative control, while PBMC stimulated with 5 μg/ml phytohemagglutinin (PHA, Sigma-Aldrich, St Louis, MO) were used as a positive control of cell reactivity. Wells containing biofilms without PBMC were also established for bacterial count. Following incubation, PBMC suspensions, collected from six wells of each experimental condition were pooled. A 50 μl aliquot was taken from each sample for cell viability assessment prior to centrifugation at 500 × g for 5 min. Supernatants were collected from each condition, sterile filtered (0.22 μm), aliquoted, and stored at −80°C for further assays (i.e., cytokine determination and biofilm stimulation). The PBMC pellet was resuspended in PBS and cell surface staining was performed.

### Evaluation of Cell Viability by Trypan Blue Dye Exclusion Test

Viability of PBMC was assessed after 24 h co-culture with *P*. *aeruginosa* biofilms by the trypan blue dye exclusion test. To this aim, PBMC incubated alone, co-cultured with preformed biofilms, or stimulated with PHA were diluted 5 fold with 0.4% trypan blue, added into a Burker counting chamber (Sigma-Aldrich), and observed under 400 × magnification with light microscope (Olympus CH20BIMF200, Olympus optical Ltd, Japan). Alive (clear) and dead (blue) cell numbers were assessed independently by two operators and the counts obtained from six fields were averaged.

### Immunofluorescence Staining for Cell Surface Markers

PBMC incubated with and without biofilm or stimulated with PHA were subjected to surface staining for activation and cell surface markers. Two- or three-color immunofluorescence staining was performed as previously described (Esin et al., 2013). Briefly, the cells were washed with PBS by centrifugation at 500 × g for 5 min at 4°C and incubated with saturating amounts of monoclonal antibodies (MAbs) directed against cell surface or activation markers for 30 min at 4°C.

The following MAbs were used for the staining: fluorescein isothiocyanate (FITC)-conjugated anti-CD3 or anti HLA-DR; PE-conjugated anti-CD14, anti-CD19, or anti-CD69 (Miltenyi Biotec, Bergisch Gladbach, Germany); rhodamine-PE-cyanin 5.1(PC5)-conjugated anti-CD56 MAb (Beckman Coulter srl, Milan, Italy). Isotype matched mouse immunoglobulin G (IgG) Mabs (Miltenyi Biotec) were used as negative controls.

Following staining and a wash with PBS, the cells were fixed with 1% paraformaldehyde in PBS (Sigma-Aldrich) for 24 h at 4°C. After a wash with PBS, at least 50000 events were acquired ungated in a BD Accuri C6 flow cytometer (BD Biosciences, San Jose, CA, USA). BD Accuri C6 software (BD Biosciences) was used for computer-assisted analyses. For the analyses, cells of interest were selected by a widely set gate on a two-parameter plot of side scatter (SSC) vs. forward-angle scatter (FSC) excluding debris and events not of interest; among these cells the percentage and/or mean fluorescence intensity (MFI) of the surface markers were calculated according to following panel combinations: negative control-FITC/-PE/-PC5; CD3-FITC/CD69-PE/CD56-PC5; HLA-DR-FITC/CD19-PE.

### Determination of Cytokines in Culture Supernatants

The levels of a panel of cytokines (IL-1β, IL-6, IL-10, IL-4, IL-8, IFN-γ, TNF-α) present in the co-culture supernatants were determinated by a flow cytometer based multibead capture assay (LEGENDplex^TM^Multi-Analyte Flow Assay Kit, BioLegend Inc., San Diego, CA, USA) according to manufacturer's instructions. Sensitivities of the assay were as follows: IL-1β, 0.65 ± 0.47 pg/ml; IL-4, 0.97 ± 0.83 pg/ml; IL-6, 0.97 ± 1.46 pg/ml; IL-8, 1.90 ± 0.65 pg/ml; IL-10, 0.77 ± 1.18 pg/ml; IFN-γ 0.76 ± 0.53, pg/ml; TNF-α, 0.88 ± 0.27 pg/ml. Samples were acquired in a BD Accuri C6 flow cytometer (BD Biosciences), analyzed with LegendPlex v8.0 Software (BioLegend Inc.), and referred to a standard curve. Results were expressed as pg/ml or ng/ml depending on the cytokine.

### Incubation of *P. aeruginosa* Biofilms With Supernatants From PBMC-Biofilm Co-culture

Preformed 24 h-old *P*. *aeruginosa* ATCC 27853 biofilms were washed three times with PBS. Supernatants, obtained from wells of PBMC co-cultured with *P*. *aeruginosa* biofilms (see “PBMC:biofilm co-culture” above), were diluted 1:1 with fresh complete RPMI and added to the biofilms. Following a 24 h incubation at 37°C, the number of biofilm-associated cells was evaluated by CFU count, as described before. In some experiments, the effect of PBMC-biofilm supernatants derived from two different donors were assessed against 24 h-old biofilms of *P*. *aeruginosa* PAO1 strain and two *P*. *aeruginosa* clinical isolates (PA-AF and PA-CF).

### Statistical Analysis

The statistical significance of the data was determined by Student's *t*-test for paired samples or by non-parametric Wilcoxon signed-rank test. For multiple comparisons ANOVA for matched samples followed by Tukey-Kramer multiple comparisons test were used. A *P*-value of < 0.05 was considered significant.

## Results

### Formation of *P. aeruginosa* Biofilms in Complete RPMI 1640 Medium at 24 and 48 h

**Figure 1:**
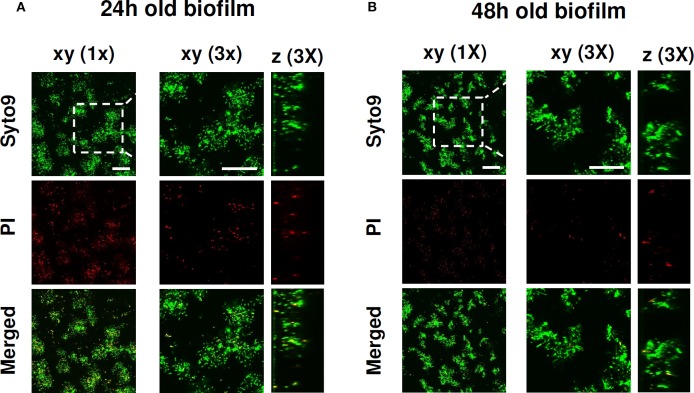
CLSM images of *P. aeruginosa* ATCC 27853 biofilm formed in RPMI medium. The viability of *P. aeruginosa* and the biofilm structure were evaluated upon **(A)** 24 and **(B)** 48 h of culture in complete RPMI 1640 at 37°C. After a wash to eliminate planktonic bacteria, *P. aeruginosa* biofilms were stained with green fluorescent labeled Syto 9 (488/500–540 nm) for alive bacteria and with red fluorescent propidium iodide (PI, 488/600–650 nm) for dead bacteria. Figure shows results from a representative experiment of the two performed. Dashed square indicates the 3x zoomed area. Scale bar = 25 μm.

The biofilm forming ability of *P*. *aeruginosa* ATCC 27853 in an eukaryotic cell-compatible medium was assessed by incubating stationary phase *P*. *aeruginosa* in complete RPMI 1640 medium. Following 24 and 48 h of incubation at 37°C biofilm, viability and structure were evaluated by CLSM analysis. As shown in Figure 1, at both 24 and 48 h, typical micro-colonies attached on the slide were observed. Moreover, Syto9/PI staining demonstrated that most of the bacterial cells within the biofilm structure were alive, confirming the ability of *P*. *aeruginosa* to successfully form biofilm in the adopted conditions.

### Evaluation of PBMC Viability Upon Incubation With Preformed *P. aeruginosa* Biofilms

**Figure 2:**
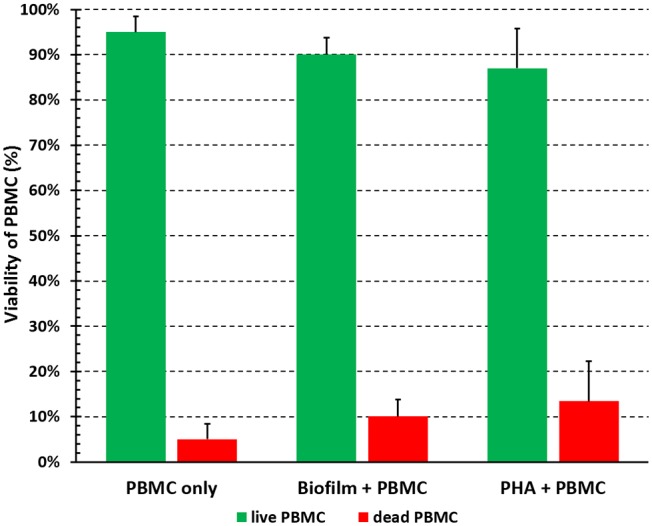
Viability of PBMC incubated with preformed *P. aeruginosa* biofilms for 24 h. PBMC were collected after 24 h of incubation in the absence (PBMC only) and in the presence (Biofilm + PBMC) of preformed biofilms, or stimulation with a mitogen (PHA + PBMC) in complete RPMI, and percentage of live and dead cells were assessed by trypan blue exclusion test. Mean values ± SEM from six independent experiments are shown.

The viability of PBMC upon incubation with preformed *P*. *aeruginosa* biofilms in complete RPMI medium was assessed by trypan blue dye exclusion test. Preliminary experiments were performed to establish an incubation time long enough to measure immune functions of PBMC with minimal cell death (data not shown). As depicted in Figure 2, PBMC incubated for 24 h with *P*. *aeruginosa* biofilms were 90 ± 3% alive and this was comparable to cells stimulated with a mitogen (PHA, 87 ± 9%). The viability of PBMC incubated alone was 95 ± 4%, indicating that the conditions adopted for *in vitro* PBMC:biofilm co-cultures allowed to keep the human cells alive at acceptable levels for 24 h.

### Activation of PBMC Subsets Upon 24 h Co-culture With *P. aeruginosa* Biofilms

After gradient separation and before adding on biofilms, the PBMC typically consisted of 72.8 ± 2.9% CD3^+^ T cells, 8.5 ± 0.8% CD3^−^CD56^+^ NK cells, 9.7 ± 4.4% CD14^+^ monocytes and 9.0 ± 2.0% CD19^+^ B cells. To evaluate whether PBMC:biofilm co-culture induced activation of PBMC, cells were harvested at 24 h and stained with MAbs directed against cell surface subset markers (CD3^+^, T cells; CD56^+^CD3^−^, NK cells; CD19^+^, B lymphocytes; CD14^+^, monocytes) and the early activation marker CD69 (for T and NK cells) or for HLA-DR (for B lymphocytes).

**Figure 3:**
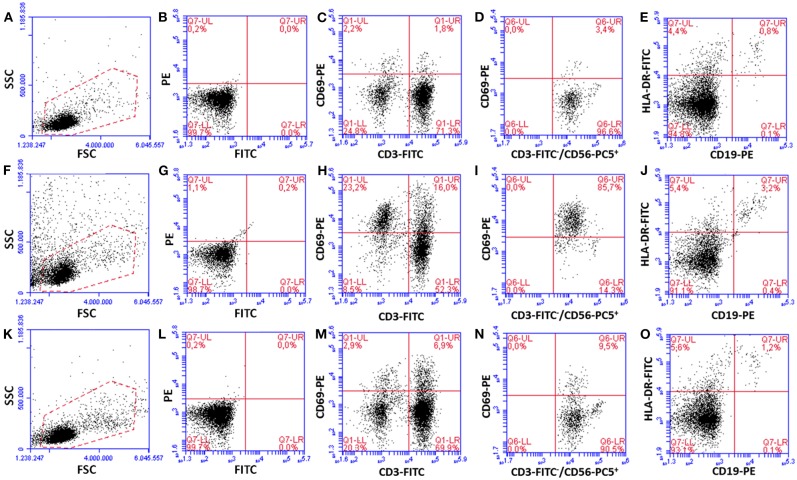
Activation of PBMC subsets upon 24 h of incubation with preformed *P. aeruginosa* biofilms. Dot plots from a representative experiment are shown. PBMC incubated alone **(A–E)**, co-cultured for 24 h with preformed *P. aeruginosa* biofilms **(F–J)**, and incubated with a mitogen [PHA, **(K–O)**] were harvested and surface stained with MAbs against cell subset markers (CD3^+^ T cells, CD56^+^CD3^−^ NK cells, CD19^+^ B cells), activation marker CD69 (for T and NK cells), and HLA-DR. **(A,F,K)** A gate (red dotted lines) which includes resting and activated live PBMC was set for the analyses. **(B,G,L)** Isotype matched mouse IgGs, used as negative controls (isotype control for PC5 is not shown). **(C,H,M)** T cells, **(D,I,N)** NK cells (CD69 staining on CD56^+^CD3^−^ cells gated from CD3 vs. CD56 plots are shown), **(E,J,O)** B cells.

As an average of seven different donors, T and NK cells co-cultured for 24 h with *P*. *aeruginosa* mature biofilms expressed the early activation marker CD69 at statistically higher levels as compared to PBMC incubated alone (CD69^+^ CD3^+^ T cells, 11.5 ± 1.6% vs. 1.6 ± 0.3% *P* < 0.001, Student's t-test for paired samples; CD69^+^ CD3^−^ CD56^+^ NK cells, 6.4 ± 2.3% vs. 0.6 ± 0.2% *P* < 0.05, respectively; see Figure 3 for data from a representative donor). As professional antigen presenting cells, B lymphocytes constitutively express HLA-DR marker whose intensity increases upon antigen-driven activation (Liu et al., 2017). Although the percent of B lymphocytes incubated with *P*. *aeruginosa* biofilms was significantly higher than that of PBMC incubated alone, no significant increase in the HLA-DR mean fluorescence intensity was observed on CD19^+^ cells upon co-culture with *P*. *aeruginosa* biofilms or incubation with PHA (Supplementary Figure 1).

**Figure 4:**
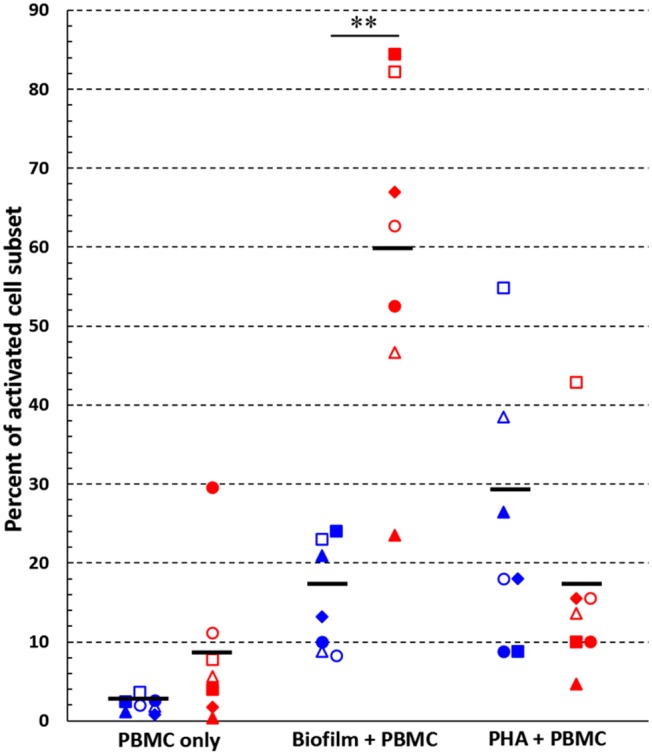
Activated cells among T and NK cell subsets. Percentages of activated (CD69^+^) cells within T cell- (blue symbols) and NK cell (red symbols) -subsets, respectively, were calculated following 24 h incubation of PBMC alone, with preformed *P. aeruginosa* biofilms, or with a mitogen (PHA). ^**^P < 0.01, Student's *t*-test for paired samples, *n* = 7. Black lines indicate mean values. Each symbol indicates a different donor/experiment.

Next, we analyzed the percentage of activated cells within each cell subset (Figure 4). Interestingly, upon 24 h of co-culture with preformed *P*. *aeruginosa* biofilms, 17 ± 3% of all T cells were activated whereas the percentage reached the value of 60 ± 8% in the case of CD56^+^CD3^−^ NK cells (*P* < 0.05). In some donors, as much as 85.7% of CD56^+^CD3^−^ NK cells were activated (Figure 3I). Therefore, the NK cell subset represented the most activated cell subset upon stimulation with *P*. *aeruginosa* biofilms. Only 3 ± 1% of T cells and 9 ± 4% of NK cells, were activated when the PBMC were incubated alone (Figure 4). As expected, the stimulation of PBMC with PHA resulted in an increase of activated cells that were mainly expressing the T cell marker CD3^+^ (Figures 3M, 4). In this case, 29 ± 8% of T cells were activated, while the mean percentage of the NK cells was 17 ± 7% (Figure 4).

In all experimental conditions, CD14^+^ cells among harvested PBMC were <0.5%, suggesting that monocytes adhered or remained entrapped within the biofilm during the incubation or were lysed in the attempt to ingest biofilm cells.

### Cytokine Secretion Profile of PBMC Co-cultured With *P*. *aeruginosa* Biofilms

In order to establish whether cell activation resulted in functional activity of PBMC co-cultured with *P*. *aeruginosa* biofilms, we evaluated the cytokine profile (IL-1β, IL-4, IL-6, IL-8, IL-10, TNF-α, IFN-γ) in the co-culture supernatants and compared it to that of PBMC incubated alone or stimulated with PHA.

**Figure 5:**
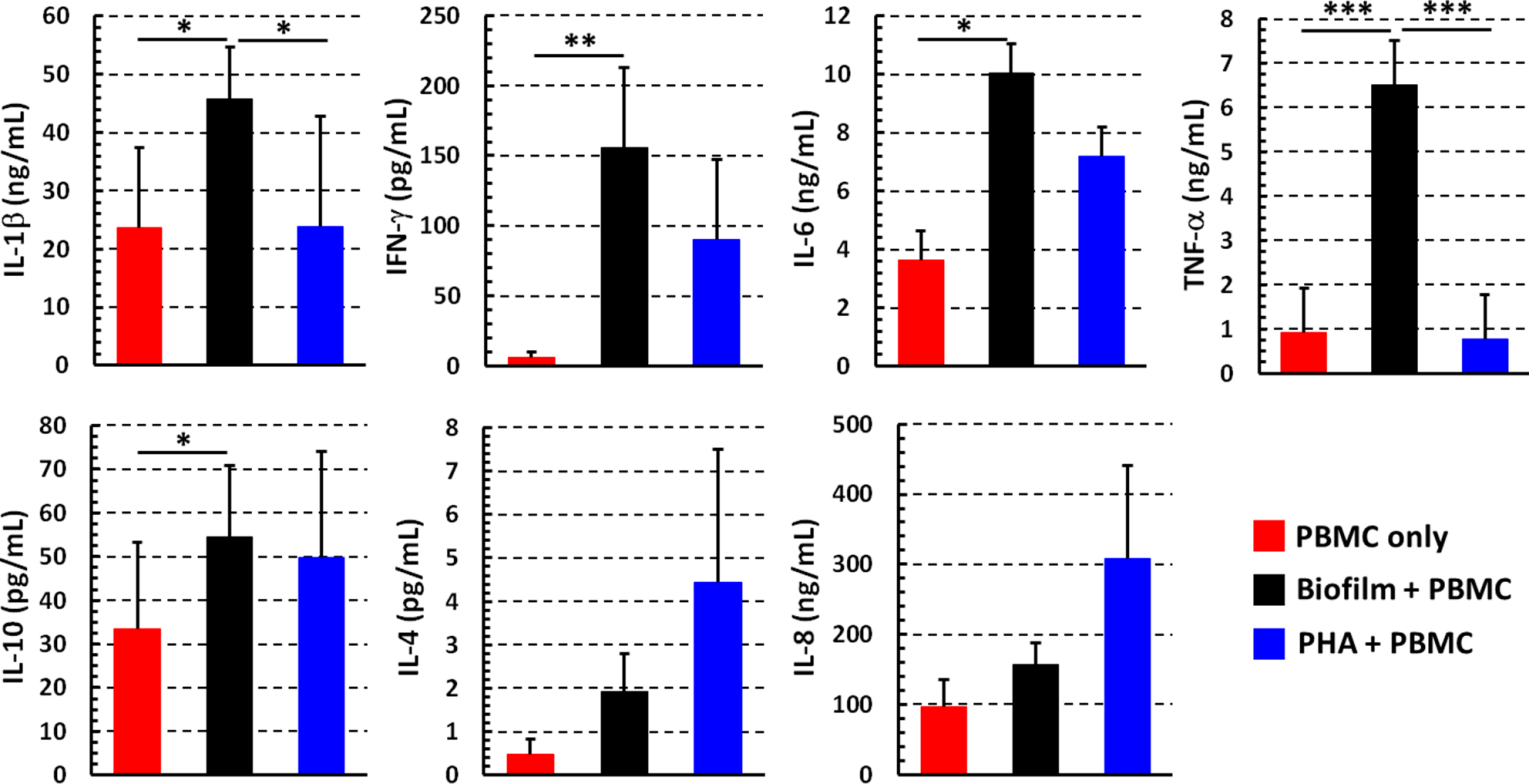
Cytokine profiles of PBMC stimulated with *P. aeruginosa* biofilm. The supernatants were collected after 24 h from wells in which PBMC incubated alone (red bars), with preformed *P. aeruginosa* biofilm (black bars), or stimulated with a mitogen (PHA, blue bars) in complete RPMI. Cytokine amount was evaluated by flow cytometer based multibead capture assay. Mean values ± SEM from 10 independent experiments are shown ^*^P < 0.05, ^**^P < 0.01, ^***^P < 0.001, ANOVA for matched samples and Tukey-Kramer multiple comparisons test.

Among the pro-inflammatory cytokines tested, IL-1β, IFN-γ, IL-6, and TNF-α were significantly increased in the supernatants of PBMC co-cultured 24 h with *P*. *aeruginosa* biofilms in comparison to PBMC incubated in the absence of bacteria, whereas there were no significant differences in the IL-8 levels (Figure 5). Interestingly, in addition to pro-inflammatory cytokines, anti-inflammatory cytokine IL-10 was also produced at relatively high levels by PBMC co-cultured in the presence of *P*. *aeruginosa* biofilms as compared to PBMC incubated alone. The amount of IL-4 secreted into culture supernatants was very low overall and without any significant differences. As expected, PHA stimulated the secretion of most of the tested cytokines from PBMC (Figure 5).

### Effect of PBMC: Biofilm Co-culture on the CFU Number of Biofilm-Associated *P. aeruginosa*

**Figure 6:**
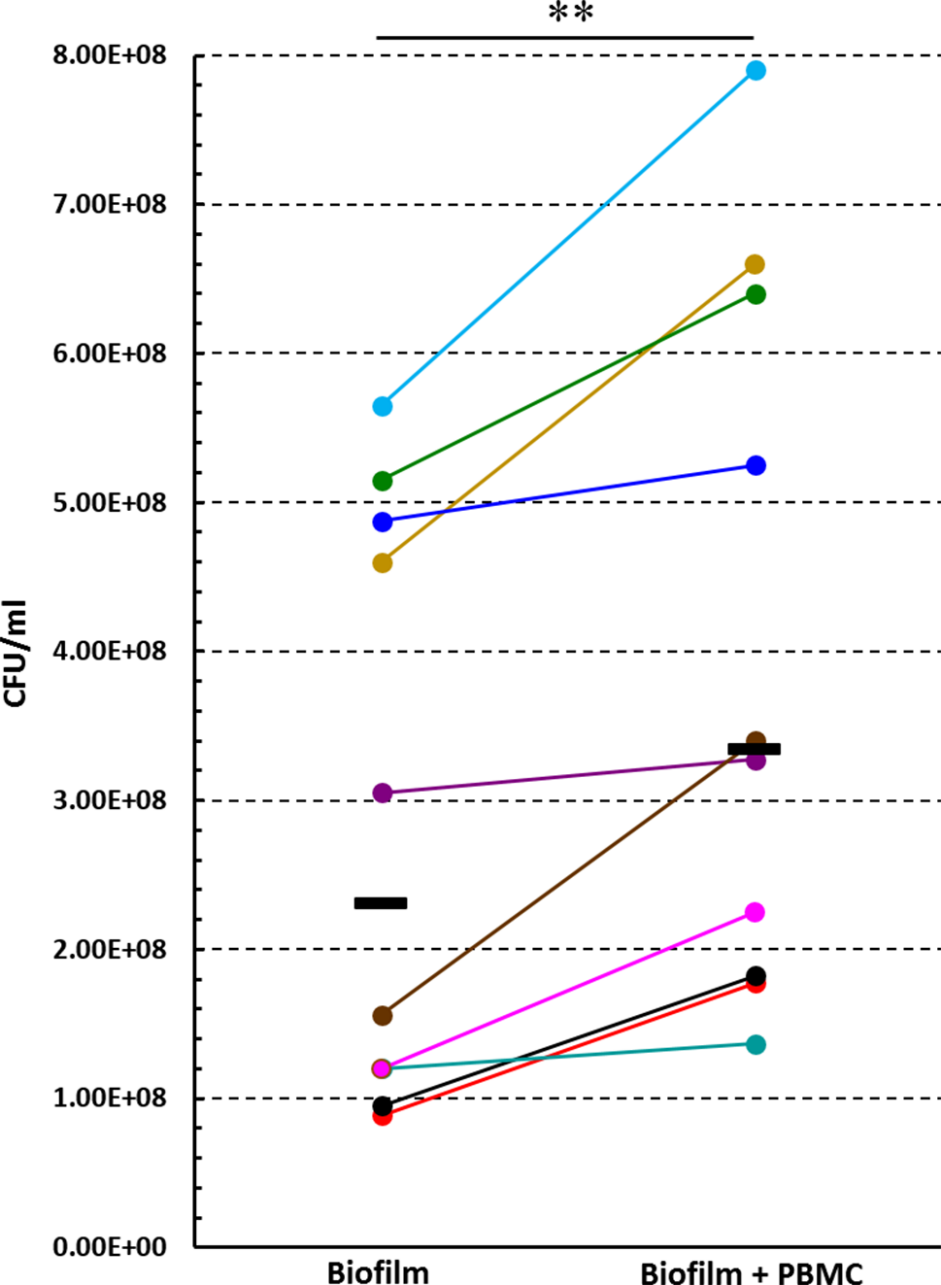
The effect of PBMC:*P. aeruginosa* biofilm co-culture on the number of *P. aeruginosa* biofilm associated bacteria. Preformed (24 h old) *P. aeruginosa* biofilms were cultured in the presence or absence of PBMC obtained from different donors for further 24 h, and the numbers (CFU) of biofilm associated bacteria were assessed. Each donor/experiment is represented by a different color. Black lines represent median values.^**^P < 0.01, Wilcoxon signed-rank test, *n* = 10.

In order to investigate whether PBMC could affect the number of biofilm-associated *P*. *aeruginosa* upon 24 h of co-culture, preformed *P. aeruginosa* biofilms (24 h old) were incubated in the presence or absence of PBMC from different donors for further 24 h. After gently washing biofilm 3 times with warm PBS to remove planktonic bacteria and PBMC without disrupting the biofilm, biofilm-associated bacteria were harvested by mechanically disrupting the biofilm and plated on TSA. Interestingly, as shown in Figure 6, a statistically higher CFU number was obtained from biofilm incubated in presence of PBMC from different donors as compared to the corresponding biofilms cultured without PBMC (*P* < 0.01), indicating that co-cultivation of *P. aeruginosa* biofilms with PBMC enhances the number of biofilm-associated bacteria.

### Effect of Supernatants From PBMC:Biofilm Co-culture on *P. aeruginosa* Biofilms

**Figure 7:**
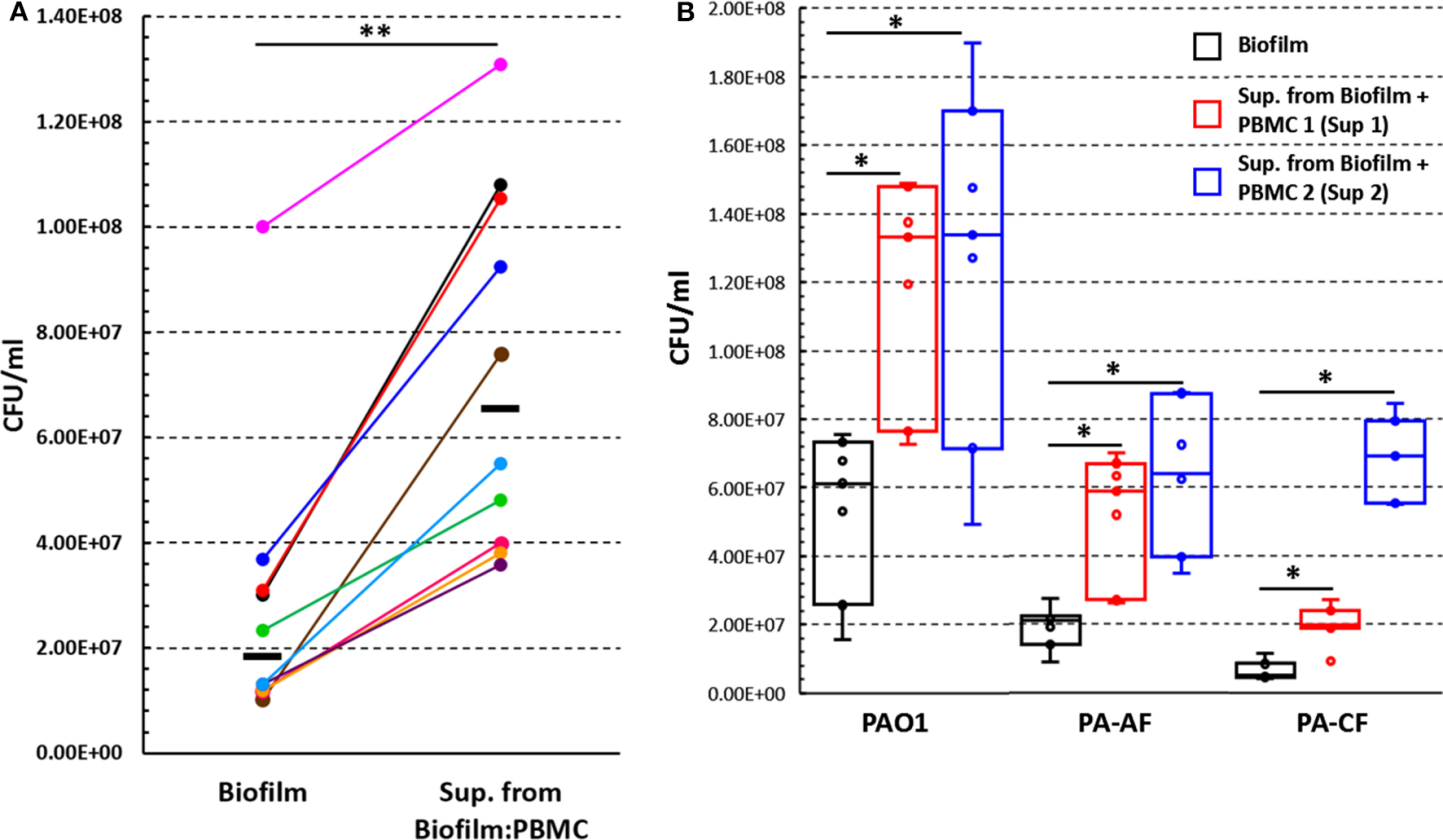
The effect of supernatants, obtained from co-culture of PBMC with 24 h old *P. aeruginosa* biofilms, on *P. aeruginosa* biofilm associated bacteria number. **(A)** Preformed *P. aeruginosa* ATCC 27853 biofilms (24 h old) were incubated for further 24 h in the presence or absence of supernatants obtained from PBMC co-cultured with preformed *P*. *aeruginosa* biofilm for 24 h (*n* = 10) and the numbers (CFU) of biofilm associated bacteria were assessed. Black lines represent median values. **(B)** Preformed biofilms of clinical *P*. *aeruginosa* strains (PAO1, PA-AF, and PA-CF) were incubated for an additional 24 h with supernatants obtained from two different donors' PBMC co-cultured with *P. aeruginosa* ATCC 27853 biofilms (*n* = 7); data are presented as the median and interquartile range. ^*^P < 0.05, ^**^P < 0.01, Wilcoxon signed-rank test.

To test the hypothesis that soluble factors produced during PBMC:biofilm co-culture could stimulate the growth of *P*. *aeruginosa* biofilms, we collected culture supernatants upon 24 h co-culture of PBMC with preformed *P*. *aeruginosa* biofilms. The supernatans, diluted 1:1 with fresh complete RPMI, were added to 24 h-old biofilms of *P*. *aeruginosa*. The number of biofilm-associated bacteria was evaluated after further 24 h incubation and compared with that of biofilms incubated without supernatants. As shown in Figure 7A, the CFU count of biofilm-associated *P*. *aeruginosa*, incubated in the presence of the supernatants, resulted two to seven times higher than that of biofilms incubated alone (*P* < 0.01).

In some experiments, we incubated 24 h old *P*. *aeruginosa* biofilms in the presence of supernatants obtained from PBMC stimulated with PHA, PBMC incubated alone, or from 24 h old *P. aeruginosa* biofilms incubated for an additional 24 h in the absence of PBMC. In all such experiments, no enhancement in the CFU of biofilm associated bacteria was observed (Supplementary Figure 2).

Next, we investigated whether the observed effect on *P*. *aeruginosa* ATCC 27853 biofilm growth was a strain specific phenomenon, or could also be true for other *P*. *aeruginosa* strains. To this aim, two different supernatants previously demonstrated to have high biofilm-enhancing ability toward the *P*. *aeruginosa* ATCC 27853 strain were selected (Sup 1 and Sup 2, Figure 7B). The two supernatants were added to 24 h old *P. aeruginosa* biofilms of PAO1 strain and two clinical isolates (PA-AF and PA-CF) and incubated for further 24 h. Although with some differences, both supernatants significantly enhanced biofilm growth of all the three strains tested as compared to the corresponding biofilms incubated in the absence of the supernatants (Figure 7B).

## Discussion

*P*. *aeruginosa* is a ubiquitous environmental bacterium that can be isolated from different habitats, including water, soil, and plants. The bacterium is also an important opportunistic human pathogen causing serious infections in immunocompromised or hospitalized patients. The large majority of infections caused by *P. aeruginosa* rely on the ability of the bacterium to form biofilm. The host response, both the innate and the acquired arms, may play a relevant role in the course of biofilm infections (Moser et al., 2017). Although a detailed knowledge of the host immune response to biofilms is still lacking, it appears that the host is mostly unable to eliminate the pathogen in the form of a biofilm. In such form, the pathogen rather persists establishing a chronic infection where the immune response, instead of being protective, may even accelerate collateral tissue damage (Moser et al., 2017). Chronic *P*. *aeruginosa* infections may persist for months to decades, as in the case of pulmonary infection in individuals with CF, during which a complex and dynamic interplay between the pathogen and the host establishes.

In the present study, in order to get further insights on the intricate relationship between *P. aeruginosa* and host immune cells, we have established an *in vitro* co-culture model of *P. aeruginosa* biofilms and PBMC from healthy blood donors. *P*. *aeruginosa* biofilms have been previously obtained *in vitro* using different types of bacteriological or synthetic media supplemented with various nutrients or growth factors (Haney et al., 2018; Wijesinghe et al., 2019). It is generally agreed that the nutrient contents of the culture medium may greatly impact the biofilm growth and development of different microbial species (Stepanović et al., 2007; Seneviratne et al., 2013; Weerasekera et al., 2016). In order to create an environment simulating the *in vivo* condition and suitable to assess immune cell responses, herein we obtained mature *P*. *aeruginosa* biofilms in RPMI 1640 supplemented with human plasma, a medium that is normally used for the growth of eukaryotic cells, but not for bacteria. Such medium resembles the composition of body fluids of the human host, as it contains high concentrations of amino acids, such as L-glutamine, L-arginine, L-asparagine, as well as vitamins and inorganic salts (Kucharíková et al., 2011). Confocal microscopy analyses, performed following 24 and 48 h of incubation of stationary phase *P*. *aeruginosa* in such medium, clearly demonstrated the presence of adherent bacterial cells organized to form characteristic microcolonies separated by empty spaces, resembling the water channels that are characteristic of *P. aeruginosa* biofilm structure (Rasamiravaka et al., 2015). Vital staining showed that most of bacterial cells were alive, confirming the ability of the RPMI 1640 medium to sustain the development of *P*. *aeruginosa* biofilms. A co-culture time of 24 h of PBMC with preformed *P. aeruginosa* biofilms was sufficient to induce cell activation with limited cell death. As an average of different donors approximately 12% of the PBMC, harvested following *P*. *aeruginosa* biofilm co-incubation, were activated T lymphocytes (CD3^+^CD69^+^ cells). Such an activation might result from a non-specific direct stimulation or from cytokines released by monocytes, but it might also be compatible with a secondary response to *P*. *aeruginosa* antigens, as the blood donors might have been sensitized by environmental exposure to *P*. *aeruginosa*, a bacterium widely distributed in the environment. Still little is known about the existence of biofilm-specific antigens and whether they are “seen” by host immune cells. Obvious candidates are components of the EPS, a highly hydrated mixture of extracellular DNA (bacteria- and host-derived), proteins, polysaccharides, and lipids whose effects on host cells appear to be of great complexity ranging from immunogenic to anti-immunogenic according to the microbial species or strains considered (Watters et al., 2016). Only few studies have investigated the T cell response to bacterial biofilms and most of them have been focused on *Staphylococcus* biofilms with some conflicting results (Prabhakara et al., 2011; Hanke et al., 2013). Early work carried out in different mouse strains intratracheally infected with *P. aeruginosa*, has demonstrated that the Th1-reacting C3H/HeN mice show a better disease outcome compared to the Th2-reacting BALB/c mice, suggesting that a Th1 response might be beneficial in chronic *P. aeruginosa* pulmonary infection (Moser et al., 1999). It is possible that different T cell subsets (Th1, Th2, Th17, Treg) take part to the response and that, similarly to other chronic infections, a balance between antigen-specific pro-inflammatory (Th1/Th17) and anti-inflammatory (Th2, Treg) T cells is required for ensuring a protective effect and a limited tissue damage (Ehlers, 1999).

A very interesting result that emerged from our study is the striking activation of CD56^+^CD3^−^ cells (NK cells) following 24 h PBMC exposure to *P*. *aeruginosa* biofilm. As an average, 60% of NK cells expressed the early activation marker CD69 upon stimulation with *P*. *aeruginosa* biofilms and the percentage reached very high levels in some donors (up to 86%). NK cells are traditionally considered as “nonspecific” or “innate” effector cells, meaning that they do not recognize bacteria in an antigen-specific manner and do not generate memory responses. Such view has been challenged over the last years when it became clear that NK cells might be endowed with those properties that for a long time have been ascribed only to cells of specific immunity (e.g., immunological memory) (Narni-Mancinelli et al., 2011; Sun et al., 2011), placing such cells at the interface between innate and adaptive immunity. The main route to the activation and functional activity of NK cells is via soluble factors (e.g., IL-12; IL-1β; TNF-α) released by accessory cells (monocytes; macrophages; dendritic cells) or T cells (e.g., IL-2). Nevertheless, we and others have provided strong evidence that NK cells express functional “pathogen recognition receptors” (PRRs) that can directly interact with “pathogen associated molecular patterns” (PAMPs), resulting in cell activation and functional activity (Chalifour et al., 2004; Esin et al., 2008, 2013). Thus, NK cell activation observed in the present study might be due to soluble factors present in the supernatants of PBMC:biofilm co-cultures, as well as to a direct recognition of yet unidentified biofilm-associated PAMPs by such cells. Putative NK cell receptors involved in the direct recognition of microbial ligands are numerous and include several members of the TLR family (e.g., TLR2, TLR4, TLR5, TLR9), intracellular receptors such as NOD2, or members of the natural cytotoxicity receptor (NCR) family (Esin and Batoni, 2015). In this regard, we have previously reported that, unlike a number of Gram-negative bacteria, a soluble form of the NCR family member NKp44 is able to bind intact *P. aeruginosa* cells suggesting the existence of putative ligands for this receptor on the bacterium surface (Esin et al., 2008). Interestingly, it has been demonstrated that a fraction of human NK cells express basal levels of TLR9 and respond to bacterial DNA containing a high frequency of unmethylated CpG motifs (Ashkar and Rosenthal, 2002; Roda et al., 2005). Since bacterial DNA is an essential component of the EPS of *P*. *aeruginosa* biofilms, it might be possible that such component may represent, among others, a biofilm-specific ligand able to activate immune NK cell response (Watters et al., 2016). Another receptor expressed on NK cells (and on other immune cells) thought to be involved in host defense against *P. aeruginosa* infection is NKG2D (Wesselkamper et al., 2008). NKG2D ligands are markedly induced by *P. aeruginosa* on pulmonary epithelial cells and the receptor seems critical for mouse protection following respiratory infection with the bacterium. Importantly, host-cell expression of NKG2D ligands was demonstrated to increase cytokine production by NK cells in response to a bacterial TLR-ligand such as LPS, suggesting a possible synergistic effect between different NK cell receptors in the response of NK cells to *P. aeruginosa* (Wesselkamper et al., 2008).

Once activated, NK cells express a variety of effector functions including cytotoxicity, production of high amounts of immunoregulatory cytokines (e.g., IFN-γ; TNF-α), production of antibacterial mediators (e.g., NO, α-defensins, granulysin), regulation of other cell type-functions and, in certain circumstances, direct bactericidal activity (Esin and Batoni, 2015) disclosing putative roles of such cells in immunity against biofilms. Of note, consistently with the large proportion of NK cell activation observed in the present study, high amount of IFN-γ were found in the supernatant of PBMC:biofilm co-culture. Interestingly, IFN-γ production by human NK cells stimulated with *P. aeruginosa* has been previously reported by Vourc'h and coworkers who demonstrated that such production is enhanced by NK priming with IL-12, but is STAT-4 pathway-independent (Vourc'h et al., 2017). The same group also demonstrated that direct bacteria-to-cell contact is necessary for IFN-γ production and the type 3-secreted exoenzyme T (Exo T) of *P. aeruginosa* is the main trigger of IFN-γ release (Vourc'h et al., 2017). Another study reported that IFN-γ release from PBMC correlates with an improved lung function in CF patients chronically infected by *P*. *aeruginosa*, suggesting a possible protective role of IFN-γ *in vivo* and a putative beneficial effect of IFN-γ treatment (Moser et al., 2000). To the best of our knowledge, massive activation of NK cells upon stimulation with *P. aeruginosa* biofilms is a finding previously not reported and point out possible roles for these cells in the response to bacterial biofilms.

Analysis of the cytokine pattern in the supernatants of PBMC:biofilm co-cultures also revealed relatively high levels of the anti-inflammatory cytokine IL-10. Such observation is consistent with the view that bacterial biofilms are able to skew the immune response toward an anti-inflammatory phenotype that promote bacterial persistence at late stage of an infection (González et al., 2018; Campoccia et al., 2019).

Interestingly, the present study demonstrated that not only *P. aeruginosa* biofilms induced activation and response of PBMC, but also the presence of PBMC or supernatant derived from PBMC:biofilm co-cultures caused a statistically significant increase of biofilm-associated *P*. *aeruginosa*. Such enhancement was observed using PBMC from different donors as well as employing different *P. aeruginosa* clinical strains suggesting that the stimulatory capacity of PBMC on *P. aeruginosa* biofilms might have important implications in the pathogenesis of *P. aeruginosa*-associated colonization of medical devices such as intravenous catheters. The increase in the number of biofilm cells in the presence of supernatants from PBMC:biofilm co-cultures suggests that soluble factors, released in the extracellular environment upon interaction of eukaryotic cells with *P. aeruginosa* biofilms, might be responsible for the observed stimulatory effect. Such effect did not seem to be due to bacterial factors (e.g., inducers of quorum sensing), in fact, biofilm supernatants obtained in the absence of PBMC did not exerted the same biofilm-enhancing effect. Neither supernatants stimulated with the mitogen PHA were able to enhance biofilm growth, suggesting that the stimulatory capacity of the supernatants was biofilm-specific and not due to a non-specific activation of host cells. It has been proposed that *P. aeruginosa* biofilms “sense” the presence of host immune components and actively react to promote its own persistence (Wu et al., 2005; Hänsch, 2012). For instance, Alhede and coworkers demonstrated that *P. aeruginosa* biofilms recognize and respond aggressively to the presence of human polymorphonuclear leukocytes (PMNs) by upregulating the synthesis of rhamnolipids, amphiphilic molecules composed of rhamnose and hydrophobic fatty acid moieties, that represent major virulence factors of the bacterium (Jensen et al., 2007; Alhede et al., 2009). There is also evidence that *P. aeruginosa* increases the formation of biofilm in the presence of PMNs (Walker et al., 2005). Possibly, cytokines or other host factors released in the supernatant upon cell activation/lysis are recognized by bacteria that implement a defensive/persistence strategy against the immune response. This hypothesis is supported by previous findings demonstrating that cytokines such as TNF-α, IL-1 or IL-6 do promote bacterial growth (Porat et al., 1991; Meduri et al., 1999; Kanangat et al., 2001) and that bacteria possess receptor-like molecules for immune mediators (Luo et al., 1993). IL-1β was shown to bind to and induce growth enhancement of *Staphylococcus aureus* biofilms (McLaughlin and Hoogewerf, 2006). Similarly, Mittal and coworkers demonstrated a significant enhancement in growth and virulence factor production when both planktonic and biofilm cells of *P. aeruginosa* were grown in the presence of supernatants containing mouse-peritoneal-macrophage secretory products (MSPs) (e.g., TNF-α, TNF-β, IL-1α, IL-β, GM-CSF, MIP-2, IL-6, IL-12, and IL-18, as well as, reactive nitrogen intermediates) (Mittal et al., 2006). Enhancement of biofilm formation by soluble factors released by adherent PBMC was also described for the fungal opportunistic pathogen *Candida albicans* (Chandra et al., 2007) suggesting that biofilm responsiveness to cytokine production may represent an adaptive response of different types of microbial pathogens to counteract the host attack. Interestingly, Wu and coworkers analyzed the ability of supernatants from antigen-stimulated T cells to induce the expression of the type I *P*. *aeruginosa* lectin (PA-I or lecA), an adhesin of *P. aeruginosa* taken by the authors as representative quorum sensing-dependent virulence determinant of this organism (Wu et al., 2005). They demonstrated that PA-I expression was increased by supernatants from activated T cell cultures and this effect was essentially ascribable to the presence of IFN-γ in the supernatants. Of note, IFN-γ-mediated increment in both transcription and translation of PA-I started at early stationary phase of growth when bacteria are considered structurally and metabolically assimilable to biofilm cells. Finally, the authors demonstrated that IFN-γ directly binds to an outer membrane protein of *P. aeruginosa* (OprF) resulting in the activation of quorum sensing, which is known to control the expression of different virulence factors including the ability to form biofilm (Wu et al., 2005). In the present study, when PBMC were co-cultured with *P*. *aeruginosa* biofilms, high amounts of IFN-γ were found in the culture supernatants, together with elevated proportion of activated NK cells that are known to be producers of IFN-γ. It is tempting to speculate that IFN-γ released by activated NK cells (and/or T cells) upon incubation with *P*. *aeruginosa* biofilms may represent a double-edged sword that is capable of expressing beneficial effector functions against biofilms, but at the same time potentially able to trigger evasion mechanisms of *P*. *aeruginosa* through activation of quorum sensing system and enhancement of biofilm formation (Moser et al., 2017).

Overall, the findings from the present study suggest that host PBMC are able to activate and produce cytokines upon interaction with *P*. *aeruginosa* biofilm. On the other hand, biofilm cells acquire the ability to grow more rapidly when challenged with mediators released from activated immune cells revealing a mechanism that may contribute to the ability of biofilms to resist clearance by host defenses and establish chronic infections. Much research effort is still needed to fully clarify the complex mechanisms and pathways that regulate the mutual interactions between the host immune system and *P*. *aeruginosa* biofilms. Previously unrecognized elements (e.g., NK cells) may participate to the host immune response to *P*. *aeruginosa* biofilms, adding complexity to the host:biofilm interaction but also paving the way for the identification of new immunotherapeutic strategies for the control of biofilm infections.

## Data Availability Statement

The datasets generated for this study are available on request to the corresponding author.

## Ethics Statement

The studies involving human participants were reviewed and approved by the ethics committee of Area Vasta Nord-Ovest (CEAVNO). The patients/participants provided their written informed consent to participate in this study.

## Author Contributions

EK, LG, GM, GB, and SE conceptualized and designed the study. EK, AB, CP, MD, GB, and SE acquired, analyzed, and interpreted the data. EK, GB, and SE drafted the article. EK, LG, AB, GM, CP, MD, GB, and SE critically revised the manuscript and approved it for publishing.

## Conflict of Interest

The authors declare that the research was conducted in the absence of any commercial or financial relationships that could be construed as a potential conflict of interest.
