# Supplementary figures and images for: In vitro Interaction of Pseudomonas aeruginosa Biofilms With Human Peripheral Blood Mononuclear Cells
Source: Front Cell Infect Microbiol. 2020 May 5;10:187. doi: 10.3389/fcimb.2020.00187 (PMC7216684; doi:10.3389/fcimb.2020.00187)

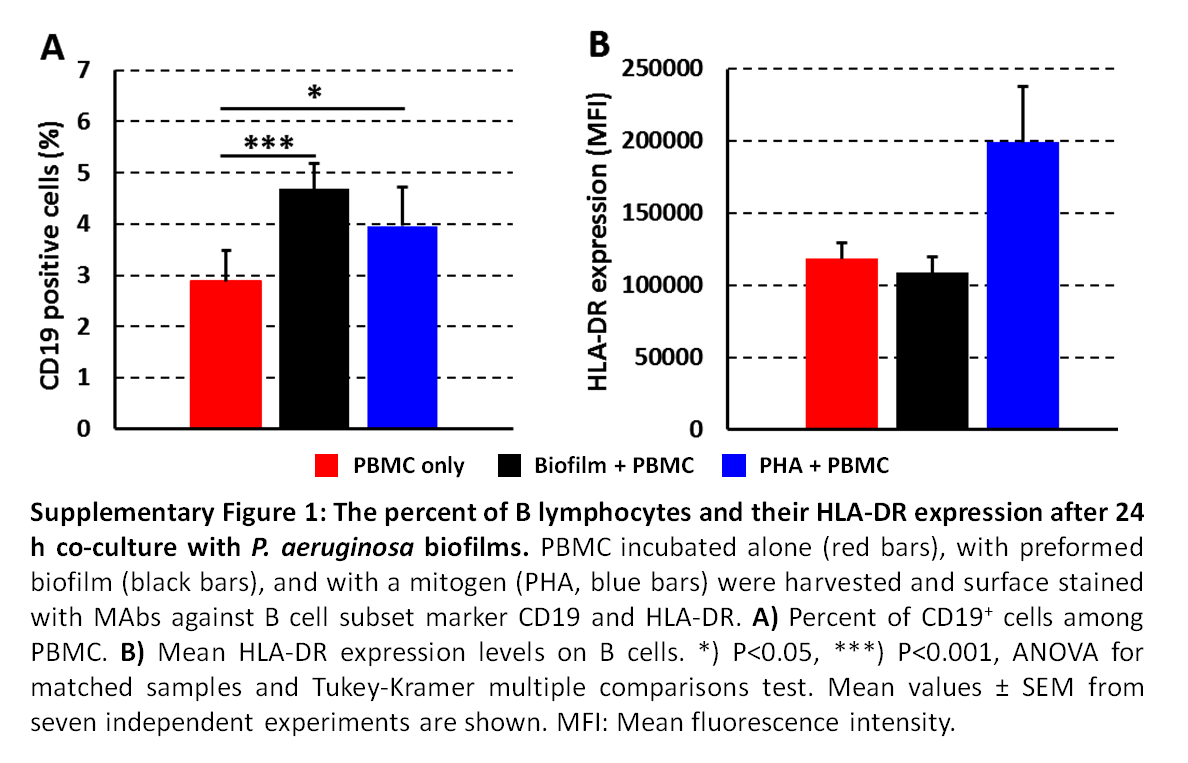

Supplement: Supplementary file 1 [file Image_1.TIF]

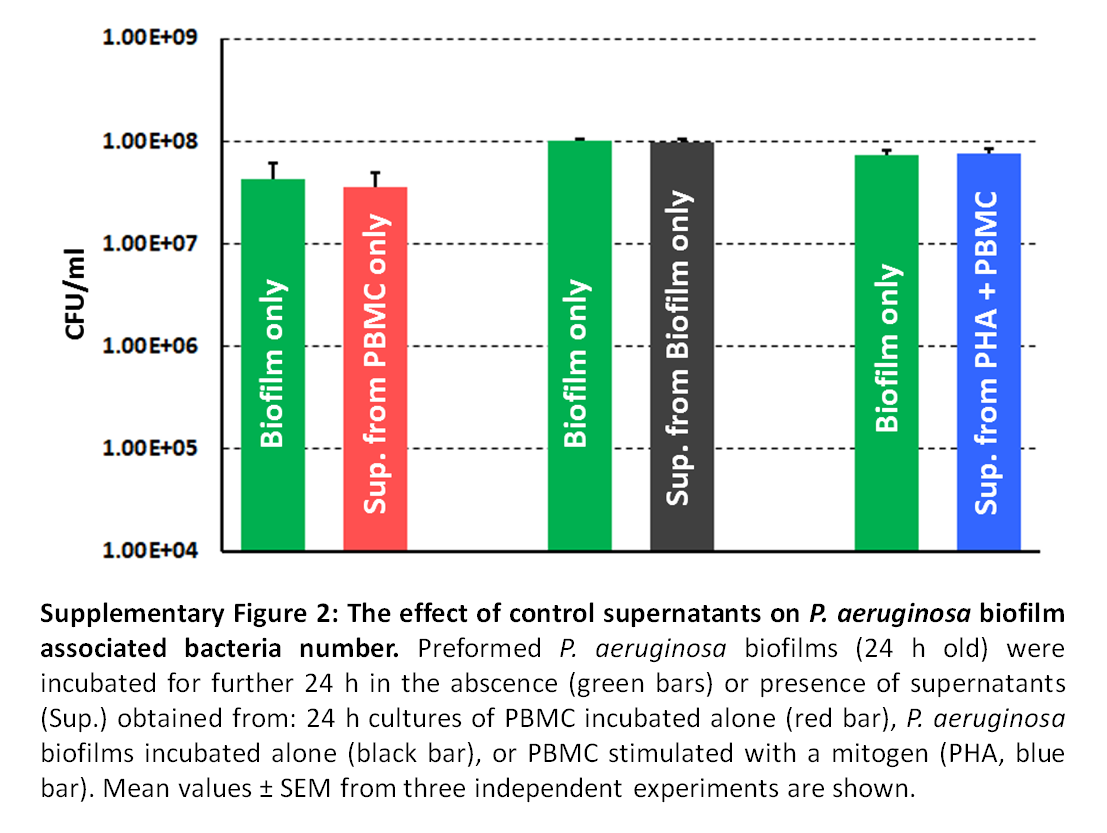

Supplement: Supplementary file 2 [file Image_2.TIF]
